# Supplementary material for: Thermodynamic principle to enhance enzymatic activity using the substrate affinity
Source: Nat Commun. 2023 Aug 24;14:4860. doi: 10.1038/s41467-023-40471-y (PMC10449852; doi:10.1038/s41467-023-40471-y)
Supplement: Supplementary file 1 — Supplementary Information [file 41467_2023_40471_MOESM1_ESM.pdf]

Supporting Information for: Thermodynamic Principle to Enhance Enzymatic Activity using the Substrate Affinity

Hideshi Ooka,[*,†] Yoko Chiba,[†,‡] and Ryuhei Nakamura[†,¶]

†*Biofunctional Catalyst Research Team*

RIKEN Center for Sustainable Resource Science (CSRS)

2-1 Hirosawa, Wako, Saitama 351-0198, Japan

‡*Faculty of Life and Environmental Science, University of Tsukuba*

1-1-1 Tennoudai, Tsukuba, Ibaraki 305-8577, Japan

¶ *Earth-Life Science Institute (ELSI), Tokyo Institute of Technology*

2-12-IE-1, Ookayama, Meguro-ku, Tokyo 152-8550, Japan

E-mail: hideshi.ooka@riken.jp

Phone: +81-(0)-48-467-9372. Fax: +81-(0)-48-462-4639

Supplementary Notes

1 Mathematical Details **3**

Appendix 1. 3

Appendix 2. 4

Appendix 3. 5

2 Influence of the Driving Force (ΔG_T) **6**

3 Influence of the Rate Constants k_1^0 and k_2^0 **8**

4 Influence of the BEP Coefficients α_1 and α_2 **9**

5 Deviations from Michaelis-Menten Kinetics **10**

5.1 Michaelis-Menten Kinetics 10

5.2 Reverse Reactions 11

5.3 Competitive Inhibition 12

5.4 Uncompetitive Inhibition 15

5.5 Substrate Inhibition 17

5.6 Allostericity 19

1 Mathematical Details

Appendix 1.

The mathematical details to obtain Eq. (6) in the main text is shown below. Based on Eq. (4), the activation barrier (E_{a1}) can be written as:

$$E_{a1} = E_{a1}^0 + \alpha_1 \Delta G_1 \tag{1}$$

Using this to substitute E_{a1} in Eq. (5) yields:

$$\begin{aligned} k_1 &= A_1 \exp \frac{-E_{a1}}{RT} && (2) \\ &= A_1 \exp \frac{-E_{a1}^0 - \alpha_1 \Delta G_1}{RT} && (3) \\ &= A_1 \exp \frac{-E_{a1}^0}{RT} \exp \frac{-\alpha_1 \Delta G_1}{RT} && (4) \\ & && (5) \end{aligned}$$

By grouping the factors independent of ΔG_1 as follows:

$$k_1^0 \equiv A_1 \exp \frac{-E_{a1}^0}{RT} \tag{6}$$

Eq. (6) in the main text can be obtained:

$$k_1 = k_1^0 \exp \frac{-\alpha_1 \Delta G_1}{RT} \tag{7}$$

Appendix 2.

The mathematical details to obtain Eq. (7) are shown below. The activation barrier for k_{1r} can be expressed in a similar way to Eq. (4) in the main text as:

$$E_{a1r} = E^0_{a1r} + \alpha_{1r}\Delta G_{1r} \tag{8}$$

By definition, $\Delta G_1 = -\Delta G_{1r}$ and $A_{1r} \exp \frac{-E^0_{a1r}}{RT} = A_1 \exp \frac{-E^0_{a1}}{RT}$. Furthermore, the thermodynamic constraint $k_1/k_{1r} = \exp \frac{-\Delta G_1}{RT}$ requires $\alpha_1 + \alpha_{1r} = 1$. Under these considerations, E_{a1r} can be expressed as:

$$E_{a1r} = E^0_{a1r} + (\alpha_1 - 1)\Delta G_1 \tag{9}$$

Therefore, based on the Arrhenius equation, k_{1r} can be expressed as:

$$\begin{aligned}
k_{1r} &= A_{1r} \exp \frac{-E_{a1r}}{RT} && (10)\\
&= A_{1r} \exp \frac{-E^0_{a1r} - (\alpha_1 - 1)\Delta G_1}{RT} && (11)\\
&= A_{1r} \exp \frac{-E^0_{a1r}}{RT} \exp \frac{(1-\alpha_1)\Delta G_1}{RT} && (12)\\
&= k^0_1 g_1^{1-\alpha_1} && (13)
\end{aligned}$$

This is Eq. (7) in the main text.

Appendix 3.

The mathematical details to obtain Eq. (8) are shown below. The activation barrier for k_2 can be expressed in a similar way to Eq. (4) in the main text as:

$$E_{a2} = E_{a2}^0 + \alpha_2 \Delta G_2 \tag{14}$$

Therefore, based on the Arrhenius equation, k_2 can be expressed as:

$$\begin{aligned} k_2 &= A_2 \exp \frac{-E_{a2}}{RT} && (15) \\ &= A_2 \exp \frac{-E_{a2}^0 - \alpha_2 \Delta G_2}{RT} && (16) \\ &= A_2 \exp \frac{-E_{a2}^0}{RT} \exp \frac{-\alpha_2 \Delta G_1}{RT} && (17) \\ &= k_2^0 g_2^{-\alpha_2} && (18) \end{aligned}$$

where k_2^0 was defined in a way similar to k_1^0 in the main text, namely $k_2^0 \equiv A_2 \exp \frac{-E_{a2}^0}{RT}$. Taking into account that $\Delta G_2 = \Delta G_T - \Delta G_1$ and therefore, $g_2 = g_T / g_1$,

$$k_2 = k_2^0 \left(\frac{g_1}{g_T} \right)^{\alpha_2} \tag{19}$$

This corresponds to Eq. (8) in the main text.

2 Influence of the Driving Force (ΔG_T)

Numerical simulations of Michaelis-Menten kinetics at varying driving forces (ΔG_T) are provided in this section. The python code provided as supplementary data can be used to generate similar plots with different parameter values.

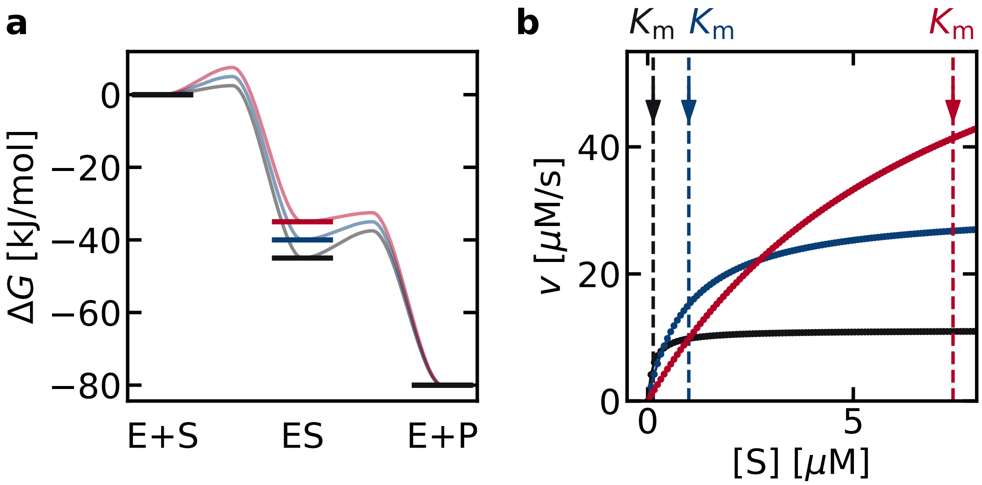

Supplementary Figure 1: Influence of the driving force at $\Delta G_T = -80$ kJ/mol and $\Delta G_1 = -35, -40, -45$ kJ/mol. All other parameters are the same as in Fig. 3 of the main text.

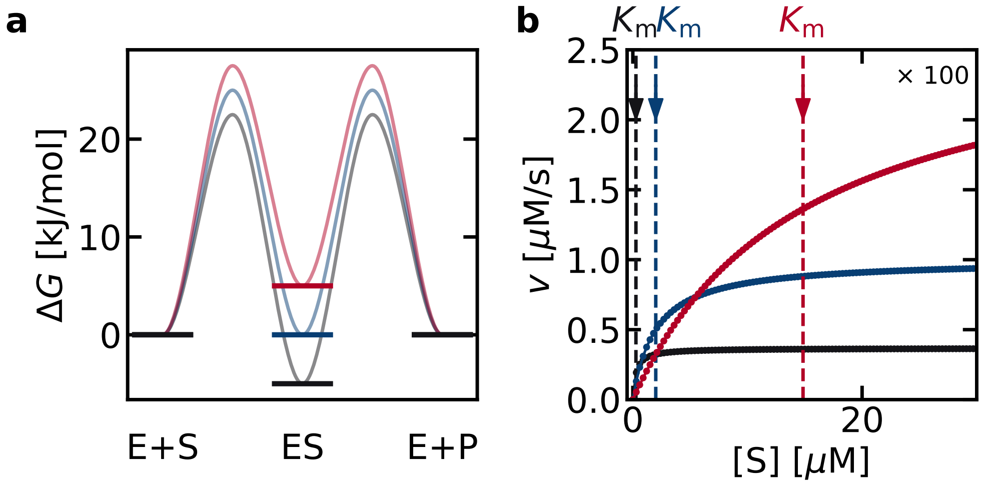

Supplementary Figure 2: Influence of the driving force at $\Delta G_T = 0$ kJ/mol and $\Delta G_1 = -5, 0, +5$ kJ/mol. All other parameters are the same as in Fig. 3 of the main text.

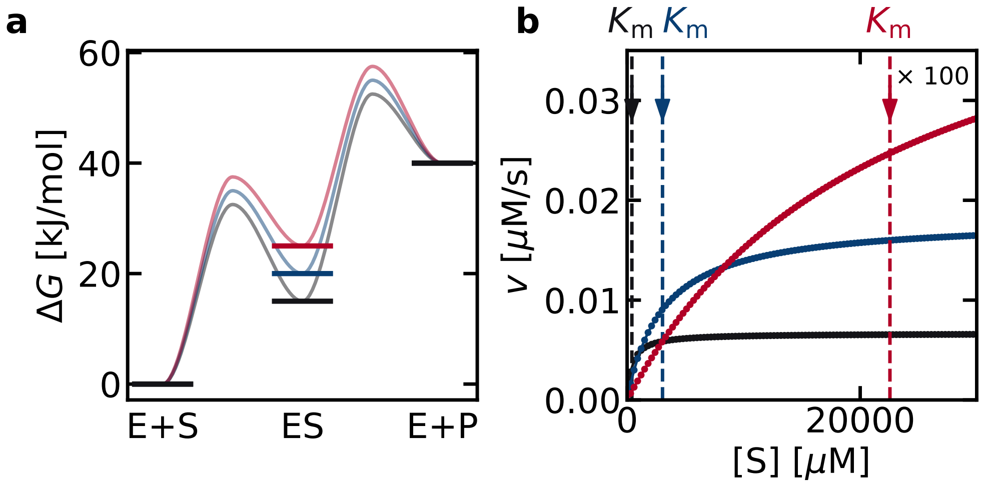

Supplementary Figure 3: Influence of the driving force at $\Delta G_T = 40$ kJ/mol and $\Delta G_1 = 15, 20, 25$ kJ/mol. All other parameters are the same as in Fig. 3 of the main text.

3 Influence of the Rate Constants k_1^0 and k_2^0

The values $k_1^0 = k_2^0 = 1$ were used in the main text, due to a necessity to set parameter values for the numerical simulations. However, the values of k_1^0 and k_2^0 do not influence the mathematical conclusion that the activity v can be maximized at $K_m = [\mathrm{S}]$, as can be seen in the simulations below.

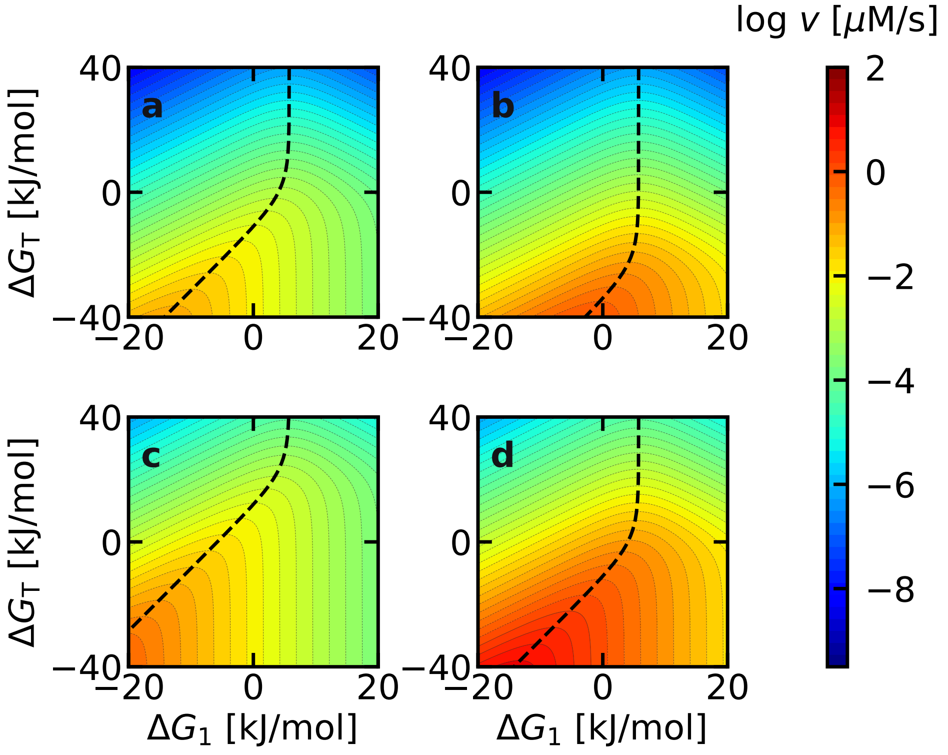

Supplementary Figure 4: Influence of k_1^0 and k_2^0 on the location of the optimum K_m. (k_1^0, k_2^0) = (0.1,0.1), (10,0.1),(0.1,10),(10,10) in panels **a** - **d**. All other parameters are the same as in Fig. 6. Regardless of the values of k_1^0 and k_2^0, maximum activity is observed at $K_m = [\mathrm{S}]$ (dashed line).

As explained in the main text, the essential requirement to achieve maximum activity is that the derivative of Eq. (13) becomes zero. This is achieved when

$$g_1(1 + K) = S \tag{20}$$

As $K_m \equiv g_1(1+K)$ (Eq. 9), the condition $K_m = [\mathrm{S}]$ guarantees that the derivative of Eq. (13)

becomes zero regardless of other parameters such as $g_1, g_T, \Delta G_1, \Delta G_T, k_1^0, k_2^0, K$. Within the scope of the model presented in the main text, the only way that the optimum K_m deviates from S is to break the assumptions leading up to Eq. (16), which is the Michaelis-Menten mechanism, and $\alpha_1 = \alpha_{1r} = \alpha_2 = 0.5$, both of which were tested directly in Fig. 6.

4 Influence of the BEP Coefficients α_1 and α_2

The values $\alpha_1 = \alpha_2 = 0.5$ were used in the main text. When this assumption is not satisfied, the optimum binding affinity deviates slightly from K_m = [S] as shown in Fig. 6d. The dependence of α values are shown in more detail below.

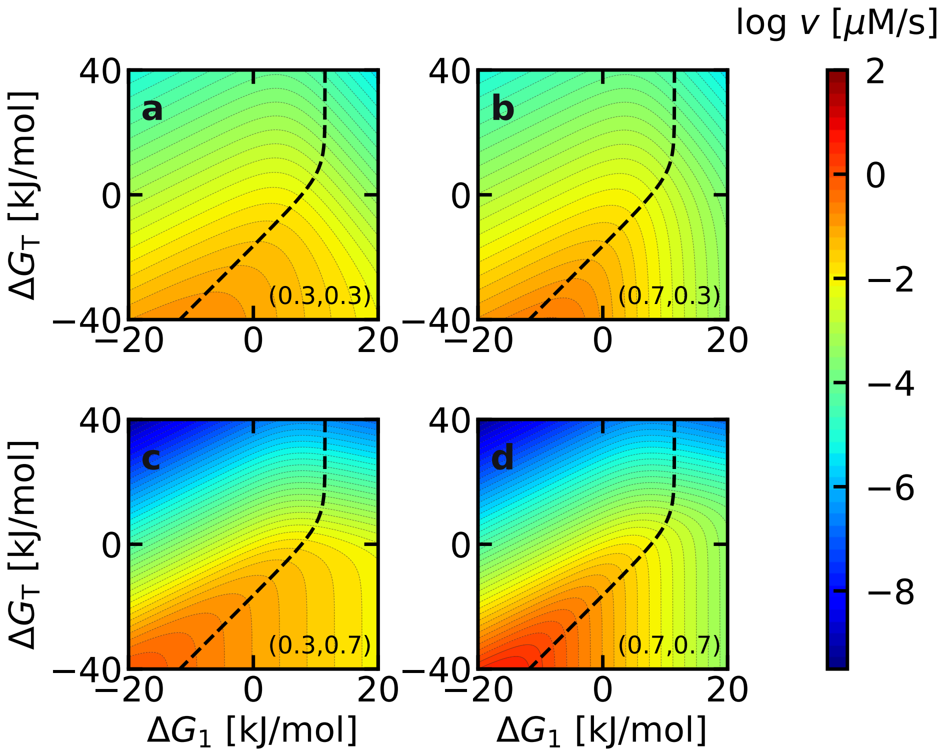

Supplementary Figure 5: Influence of α_1 and α_2 on the location of the optimum K_m. (α_1, α_2) = (0.3,0.3), (0.7,0.3),(0.3,0.7),(0.7,0.7) in panels **a** - **d**. All other parameters are the same as in Fig. 6. Regardless of the values of α_1 and α_2, maximum activity is observed near K_m = [S] (dashed line).

5 Deviations from Michaelis-Menten Kinetics

The rate laws of various chemical mechanisms will be derived in this section. We will begin our evaluation of starting from the original Michaelis-Menten equation and then evaluate how the various mechanisms will modify the rate laws.

5.1 Michaelis-Menten Kinetics

The standard Michaelis-Menten mechanism is given as follows:

$$\mathrm{E + S} \underset{k_{1r}}{\overset{k_1}{\rightleftharpoons}} \mathrm{ES} \xrightarrow{k_2} \mathrm{E + P} \tag{21}$$

The time dependence of the concentration of ES can be expressed as:

$$\frac{d[\mathrm{ES}]}{dt} = k_1[\mathrm{E}] \cdot [\mathrm{S}] - (k_{1r} + k_2)[\mathrm{ES}] \tag{22}$$

As in the main text, the concentrations of chemical species will be written upright in square brackets. Using these notations, the steady-state concentration of ES can be expressed as:

$$[\mathrm{ES}] = \frac{k_1[\mathrm{S}]}{k_{1r} + k_2}[\mathrm{E}] \tag{23}$$

$$= \frac{[\mathrm{S}]}{K_m}[\mathrm{E}] \tag{24}$$

where the definition of $K_m \equiv \frac{k_{1r}+k_2}{k_1}$ was used in the last step. As the total enzyme concentration ($[\mathrm{E_T}]$) must be constant, we obtain:

$$[\mathrm{E_T}] = [\mathrm{E}] + [\mathrm{ES}] \tag{25}$$

$$= (1 + \frac{[\mathrm{S}]}{K_m})[\mathrm{E}] \tag{26}$$

$$= \frac{[\mathrm{S}] + K_m}{K_m}[\mathrm{E}] \tag{27}$$

Therefore,

$$[\mathrm{E}] = \frac{K_m}{[\mathrm{S}] + K_m}[\mathrm{E_T}] \tag{28}$$

$$[\mathrm{ES}] = \frac{[\mathrm{S}]}{[\mathrm{S}] + K_m}[\mathrm{E_T}] \tag{29}$$

This yields the final rate as:

$$v = k_2[\mathrm{ES}] \tag{30}$$

$$= \frac{k_2[\mathrm{S}]}{K_m + [\mathrm{S}]}[\mathrm{E_T}] \tag{31}$$

5.2 Reverse Reactions

Suppose the reverse reaction cannot be ignored ($k_{2r}[\mathrm{P}] > 0$). In that case, an additional term ($k_{1r}k_{2r}[\mathrm{P}]$) must be added to the numerator to the rate law in the main text to yield:

$$v = \frac{k_1 k_2[\mathrm{S}] - k_{1r}k_{2r}[\mathrm{P}]}{k_1[\mathrm{S}] + k_{1r} + k_2 + k_{2r}[\mathrm{P}]}[\mathrm{E_T}] \tag{32}$$

$$= \frac{k_1^0 k_2^0/\sqrt{g_T}[\mathrm{S}] - k_1^0 k_2^0\sqrt{g_T}[\mathrm{P}]}{k_1^0/\sqrt{g_T}[\mathrm{S}] + k_1^0\sqrt{g_1} + k_2^0\sqrt{\frac{g_1}{g_T}} + k_2^0\sqrt{\frac{g_T}{g_1}}[\mathrm{P}]}[\mathrm{E_T}] \tag{33}$$

$$= \frac{k_2^0}{\sqrt{g_T}} \frac{[\mathrm{S}] - g_T[\mathrm{P}]}{([\mathrm{S}] + Kg_T[\mathrm{P}])/\sqrt{g_1} + (1+K)\sqrt{(g_1)}} \tag{34}$$

Setting the denominator as f, we have

$$\frac{df}{dg_1} = -0.5g_1^{-1.5}([\mathrm{S}] + Kg_T[\mathrm{P}]) + 0.5g_1^{-0.5}(1+K) = 0 \tag{35}$$

$$\Leftrightarrow g_1(1+K) = [\mathrm{S}] + Kg_T[\mathrm{P}] \tag{36}$$

indicating that $K_m = [\mathrm{S}] + Kg_T[\mathrm{P}]$ is the condition for maximum activity. When the forward reaction is favored ($\Delta G_T < 0 \leftrightarrow g_T \ll 1$), the optimum K_m converges the the substrate concentration [S].

5.3 Competitive Inhibition

Competitive inhibition occurs when an inhibitor competes for the active site of the enzyme via the following reaction:

$$\mathrm{E} + \mathrm{I} \underset{k_{ir}}{\overset{k_i}{\rightleftharpoons}} \mathrm{EI} \tag{37}$$

Let $K_i \equiv k_{ir}/k_i$ denote the extend of inhibition. Then, the steady-state concentration of EI can be expressed as:

$$[\mathrm{EI}] = \frac{[\mathrm{I}]}{K_i}[\mathrm{E}] \tag{38}$$

$$= \gamma[\mathrm{E}] \tag{39}$$

where we have set $\gamma \equiv \frac{[\mathrm{I}]}{K_i}$. In this case, the total enzyme concentration can be expressed as:

$$[\mathrm{ET}] = [\mathrm{E}] + [\mathrm{ES}] + [\mathrm{EI}] \tag{40}$$

$$= (1+\gamma)[\mathrm{E}] + [\mathrm{ES}] \tag{41}$$

$$= (1+\gamma+\frac{[\mathrm{S}]}{K_m})[\mathrm{E}] \tag{42}$$

Note that the steady-state concentration of ES was expressed using Eq. 23. Based on the above, [E] and [ES] can be expressed as follows:

$$[\mathrm{E}] = \frac{(1+\gamma)K_m}{(1+\gamma)K_m + [\mathrm{S}]}[\mathrm{E_T}] \tag{43}$$

$$[\mathrm{ES}] = \frac{[\mathrm{S}]}{(1+\gamma)K_m + [\mathrm{S}]}[\mathrm{E_T}] \tag{44}$$

Therefore, the enzymatic activity can be expressed as:

$$
\begin{aligned}
v &= k_2[\mathrm{ES}] && (45)\\
&= \frac{k_2[\mathrm{S}]}{(1+\gamma)K_m + [\mathrm{S}]}[\mathrm{E_T}] && (46)
\end{aligned}
$$

This rate expression is similar to the case without inhibition (Eq. 31). The only difference is that there is an extra term $(1+\gamma)$ before K_m, which will shift the optimum value of K_m to $[\mathrm{S}]' \equiv [\mathrm{S}]/(1+\gamma)$

In order to compare the influence of competetive inhibition, let us calculate the relative rates at $K_m = [\mathrm{S}]$ and $K_m = [\mathrm{S}']$. In the case of $K_m = [\mathrm{S}]$,

$$
\begin{aligned}
v_{K_m=[\mathrm{S}]} &= \frac{k_2[\mathrm{S}]'}{[\mathrm{S}] + [\mathrm{S}]'}[\mathrm{E_T}] && (47)\\
&= \frac{k_2(1+\gamma)}{2+\gamma}[\mathrm{E_T}] && (48)\\
&= \frac{k_2(1+\gamma)}{2+\gamma}[\mathrm{E_T}] && (49)
\end{aligned}
$$

On the other hand, in the case of $K_m = [\mathrm{S}]'$

$$
\begin{aligned}
v_{K_m=[\mathrm{S}]'} &= \frac{k_2[\mathrm{S}]'}{[\mathrm{S}]' + [\mathrm{S}]'}[\mathrm{E_T}] && (50)\\
&= \frac{1}{2}k_2[\mathrm{E_T}] && (51)
\end{aligned}
$$

It is tempting to divide Eq. 51 with Eq. 49 directly to obtain a relative activity of

$$
\frac{v_{K_m=[\mathrm{S}]}}{v_{K_m=[\mathrm{S}]'}} = \frac{2(1+\gamma)}{2+\gamma} \qquad (52)
$$

However, this approach is flawed. For example, Eq. 52 suggests that at strong inhibition $(\gamma \to \infty)$, $\frac{v_{K_m=[\mathrm{S}]}}{v_{K_M=[\mathrm{S}]'}} > 1$. This cannot be correct, as the activity should be maximized at $K_m = [\mathrm{S}]'$. The main problem is that k_2 is dependent on K_m, and therefore, the k_2 in Eq. 51

and Eq. 49 should not have been canceled out.

In order to take the relationship between K_m and k_2 into consideration, we have used the notations in the main text ($K_m = g_1(1+K), k_2 = k_2^0\sqrt{\frac{g_1}{g_T}}$). When $K_m = [\mathrm{S}]$, $g_1 = \frac{[\mathrm{S}]}{1+K}$. Therefore, Eq. 49 becomes

$$v_{K_m=[\mathrm{S}]} = \frac{1+\gamma}{2+\gamma} k_2^0 \sqrt{\frac{[\mathrm{S}]}{(1+K)g_T}} [\mathrm{E_T}] \tag{53}$$

Similarly, when $K_m = [\mathrm{S}]'$, $g_1 = \frac{[\mathrm{S}]'}{1+K}$. Therefore, Eq. 51 becomes

$$v_{K_m=[\mathrm{S}]'} = \frac{1}{2} k_2^0 \sqrt{\frac{[\mathrm{S}]'}{(1+K)g_T}} [\mathrm{E_T}] \tag{54}$$

Finally, dividing Eq. 54 with Eq. 53 gives the following expression of relative activity:

$$\frac{v_{K_m=[\mathrm{S}]}}{v_{K_m=[\mathrm{S}]'}} = \frac{2(1+\gamma)}{2+\gamma} \sqrt{\frac{[\mathrm{S}]'}{[\mathrm{S}]}} \tag{55}$$

$$= \frac{2(1+\gamma)}{2+\gamma} \sqrt{\frac{1}{1+\gamma}} \tag{56}$$

$$= \frac{2\sqrt{1+\gamma}}{2+\gamma} \tag{57}$$

Note that based on the relationship between the arithmetic and geometric mean,

$$\frac{1+(1+\gamma)}{2} \geq \sqrt{1 \cdot (1+\gamma)} \tag{58}$$

$$\tag{59}$$

Therefore,

$$\frac{v_{K_m=[\mathrm{S}]}}{v_{K_m=[\mathrm{S}]'}} = \frac{2\sqrt{1+\gamma}}{2+\gamma} \leq 1 \tag{60}$$

showing that $K_m = [\mathrm{S}]'$ is indeed the optimum value of K_m under competitive inhibition.

The relative activity when γ is varied is shown below.

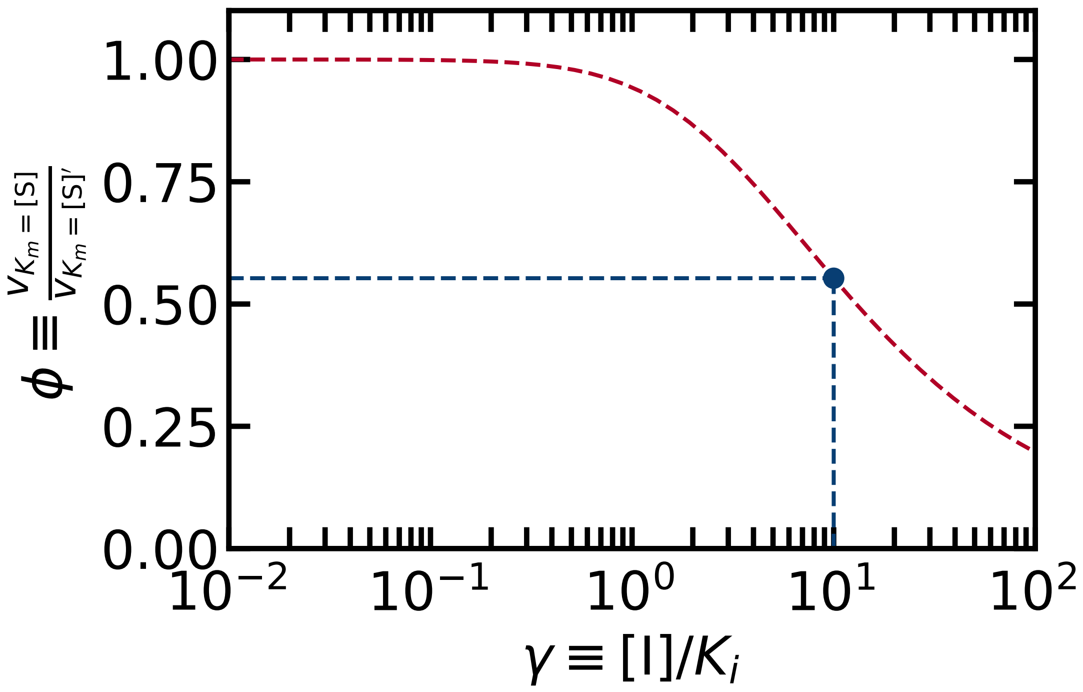

Supplementary Figure 6: Influence of competitive inhibition for γ between 10^{-3} and 10^{3}. Fig. 6b in the main text corresponds to the condition where $\gamma = 10$ (blue circle).

5.4 Uncompetitive Inhibition

In the case of uncompetitive inhibition, the following reaction decreases the concentration of the active enzyme:

$$\mathrm{ES} + \mathrm{I} \underset{k_{ir}}{\overset{k_i}{\rightleftharpoons}} \mathrm{ESI} \tag{61}$$

As in the case of competitive inhibition, let $K_i \equiv k_{ir}/k_i$. Then, the steady-state concentration of ESI can be expressed as:

$$[\text{ESI}] = \frac{[\text{I}]}{K_i}[\text{ES}] \tag{62}$$

$$= \gamma[\text{ES}] \tag{63}$$

where we have set $\gamma \equiv \frac{[\text{I}]}{K_i}$. In this case, the total enzyme concentration can be expressed as:

$$[\text{ET}] = [\text{E}] + [\text{ES}] + [\text{ESI}] \tag{64}$$

$$= [\text{E}] + (1+\gamma)[\text{ES}] \tag{65}$$

$$= (1 + \frac{[\text{S}]}{K_m}(1+\gamma))[\text{E}] \tag{66}$$

Therefore,

$$[\text{E}] = \frac{K_m}{(1+\gamma)[\text{S}] + K_m}[\text{E}_\text{T}] \tag{67}$$

$$[\text{ES}] = \frac{(1+\gamma)[\text{S}]}{(1+\gamma)[\text{S}] + K_m}[\text{E}_\text{T}] \tag{68}$$

This yields the final rate as:

$$v = k_2[\text{ES}] \tag{69}$$

$$= \frac{k_2(1+\gamma)[\text{S}]}{(1+\gamma)[\text{S}] + K_m}[\text{E}_\text{T}] \tag{70}$$

$$= \frac{k_2[\text{S}]'}{K_m + [\text{S}]'}[\text{E}_\text{T}] \tag{71}$$

where the final expression was obtained by setting $[\text{S}]' \equiv (1+\gamma)[\text{S}]$.

This expression is mathematically equivalent to the rate expression without inhibition (Eq. 31) except that $[\text{S}]'$ is used in place of [S]. As the derivations in the main text show that the maximum rate of Eq. 31 is given by $K_m = [\text{S}]$, the maximum rate for Eq. 71 is given by

$K_m = [\mathrm{S}]'$. Therefore, the maximum rate in the case of uncompetive inhibition is given by:

$$K_m = [\mathrm{S}]' \tag{72}$$

$$= (1+\gamma)[\mathrm{S}] \tag{73}$$

The dependence on γ has the exact same expression in the case of competitive inhibition (Eq. 60).

5.5 Substrate Inhibition

In the case of substrate inhibition, the reaction is inhibited when the substrate binds excessively to the enzyme. For example, a second substrate molecule may bind to the enzyme-substrate complex ($\mathrm{ES_2}$), according to the following reaction:

$$\mathrm{ES} + \mathrm{S} \underset{k_{ir}}{\overset{k_i}{\rightleftharpoons}} \mathrm{ES_2} \tag{74}$$

When $[\mathrm{ES_2}]$ is in steady-state, the following equation holds:

$$k_i[\mathrm{ES}] \cdot [\mathrm{S}] = k_{ir}[\mathrm{ES_2}] \tag{75}$$

$$\tag{76}$$

Defining $K_i \equiv k_{ir}/k_i$ gives the following expression:

$$[\mathrm{ES_2}] = \frac{[\mathrm{S}]}{K_i}[\mathrm{ES}] \tag{77}$$

$$= \gamma[\mathrm{ES}] \tag{78}$$

where $\gamma \equiv \frac{[\mathrm{S}]}{K_i}$. As the total amount of the enzyme ($[\mathrm{E_T}]$) must remain constant, the contribution from $[\mathrm{ES_2}]$ effectively serves to decrease the concentration of ES which can be used to form the product. Namely,

$$[\mathrm{E_T}] = [\mathrm{E}] + [\mathrm{ES}] + [\mathrm{ES_2}] \tag{79}$$

$$= [\mathrm{E}] + (1+\gamma)[\mathrm{ES}] \tag{80}$$

$$= (1 + \frac{[\mathrm{S}]}{K_m}(1+\gamma))[\mathrm{E}] \tag{81}$$

Therefore,

$$[\mathrm{E}] = \frac{K_m}{(1+\gamma)[\mathrm{S}] + K_m}[\mathrm{E_T}] \tag{82}$$

$$[\mathrm{ES}] = \frac{(1+\gamma)[\mathrm{S}]}{(1+\gamma)[\mathrm{S}] + K_m}[\mathrm{E_T}] \tag{83}$$

This yields the final rate as:

$$v = k_2[\mathrm{ES}] \tag{84}$$

$$= \frac{k_2(1+\gamma)[\mathrm{S}]}{(1+\gamma)[\mathrm{S}] + K_m}[\mathrm{E_T}] \tag{85}$$

$$= \frac{k_2[\mathrm{S}]'}{K_m + [\mathrm{S}]'}[\mathrm{E_T}] \tag{86}$$

where the final expression was obtained by setting $[\mathrm{S}]' \equiv (1+\gamma)[\mathrm{S}]$.

This expression is equivalent to competitive inhibition, because in both cases, an inhibitor decreases the concentration of ES by converting it into an inactive state. Substrate inhibition is a special case of competitive inhibition where the inhibitor is the substrate molecule itself. However, within the simplified framework of Michaelis-Menten kinetics, the identity of the inhibitor does not alter the final rate equation.

5.6 Allostericity

As a final example of deviation from Michaelis-Menten kinetics, we have considered allostericity according to the following mechanism:

$$\mathrm{E + nS} \underset{k_{1r}}{\overset{k_1}{\rightleftharpoons}} \mathrm{ES_n} \xrightarrow{k_2} \mathrm{E + nP} \tag{87}$$

This is a mechanism corresponding to positive homotropic allostericity such as in the case of hemoglobin, where the substrate is also an effector which enhances the activity.

Based on the steady-state approximation of $\mathrm{ES_n}$, the following expression can be obtained:

$$k_1[\mathrm{E}] \cdot [\mathrm{S}]^n = (k_{1r} + k_2)[\mathrm{ES_n}] \tag{88}$$

$$[\mathrm{ES_n}] = \frac{k_1}{k_{1r} + k_2}[\mathrm{E}][\mathrm{S}]^n \tag{89}$$

$$= \frac{[\mathrm{S}]^n}{K_m}[\mathrm{E}] \tag{90}$$

Eq. 90 is the same as Eq. 23 except that [S] has been replaced with $[\mathrm{S}]^n$. Therefore, the final rate equation can be obtained by replacing the [S] in Eq. 31 with $[\mathrm{S}]^n$:

$$v = \frac{k_2[\mathrm{S}]^n}{K_m + [\mathrm{S}]^n}[\mathrm{E_T}] \tag{91}$$

This equation corresponds to the famous Hill-Langmuir equation of allosteric enzymes, and the optimum K_m is given at $K_m = [\mathrm{S}]^n$, not $K_m = [\mathrm{S}]$. The difference in activity can be calculated from

$$v_{K_m=[\mathrm{S}]} = \frac{k_2[\mathrm{S}]^n}{[\mathrm{S}] + [\mathrm{S}]^n}[\mathrm{E_T}] \tag{92}$$

$$= \frac{[\mathrm{S}]^n}{[\mathrm{S}] + [\mathrm{S}]^n}\sqrt{\frac{[\mathrm{S}]}{(1+K)g_T}}k_2^0[\mathrm{E_T}] \tag{93}$$

and

$$v_{K_m=[\mathrm{S}]^n} = \frac{k_2[\mathrm{S}]^n}{[\mathrm{S}]^n + [\mathrm{S}]^n}[\mathrm{E_T}] \tag{94}$$

$$= \frac{1}{2}k_2[\mathrm{E_T}] \tag{95}$$

$$= \frac{1}{2}\sqrt{\frac{[\mathrm{S}]^n}{(1+K)g_T}}k_2^0[\mathrm{E_T}] \tag{96}$$

as

$$v_{K_m=[\mathrm{S}]}/v_{K_m=[\mathrm{S}]^n} = \frac{2[\mathrm{S}]^{\frac{1+n}{2}}}{[\mathrm{S}] + [\mathrm{S}]^n} \tag{97}$$

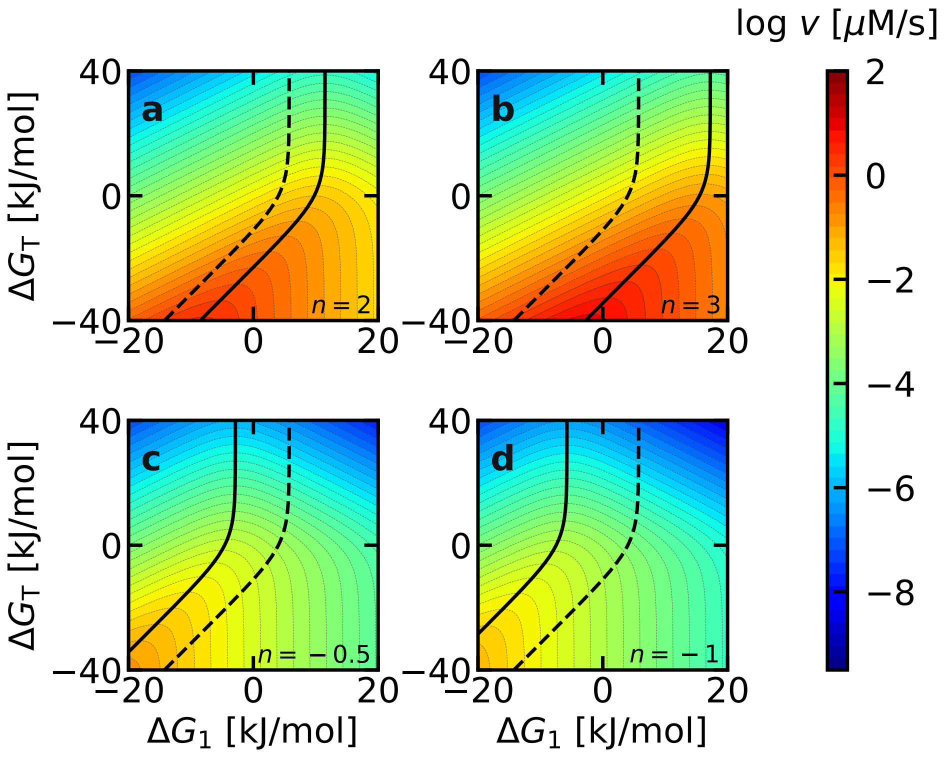

Supplementary Figure 7: Influence of allostericity with a Hill constant (n) of **a** 2, **b** 3, **c** -0.5, **d** -1. Other parameters are the same as Fig. 6 in the main text. Even under varying degrees of allostericity, the dashed line ($K_m = [\mathrm{S}]$) is located near the solid line showing the true optimum K_m ($K_m = [\mathrm{S}]^n$) and passes through the high activity region (orange).
