# Supplementary material for: Thermodynamic principle to enhance enzymatic activity using the substrate affinity
Source: Nat Commun. 2023 Aug 24;14:4860. doi: 10.1038/s41467-023-40471-y (PMC10449852; doi:10.1038/s41467-023-40471-y)
Supplement: Supplementary file 3 — Description of Additional Supplementary Files [file 41467_2023_40471_MOESM3_ESM.docx]

**Description of Additional Supplementary Files**

File Name: Supplementary Data

Description: Dataset obtained from Park et al., Nat. Chem. Biol., 2016, 12, 482–489.

File Name: Supplementary Data 2

Description: Main python code used for numerical simulations.
